# Fumarylacetoacetate Hydrolase Knock-out Rabbit Model for Hereditary Tyrosinemia Type 1*[Fn FN2]

**DOI:** 10.1074/jbc.M116.764787

**Published:** 2017-01-04

**Authors:** Li Li, Quanjun Zhang, Huaqiang Yang, Qingjian Zou, Chengdan Lai, Fei Jiang, Ping Zhao, Zhiwei Luo, Jiayin Yang, Qian Chen, Yan Wang, Philip N. Newsome, Jon Frampton, Patrick H. Maxwell, Wenjuan Li, Shuhan Chen, Dongye Wang, Tak-Shing Siu, Sidney Tam, Hung-Fat Tse, Baoming Qin, Xichen Bao, Miguel A. Esteban, Liangxue Lai

**Affiliations:** From the a CAS Key Laboratory of Regenerative Biology, Joint School of Life Sciences, Guangzhou Institutes of Biomedicine and Health, Chinese Academy of Sciences, Guangzhou 510530, China, and Guangzhou Medical University, Guangzhou 511436, China,; b CAS Key Laboratory of Regenerative Biology and Guangdong Provincial Key Laboratory of Stem Cells and Regenerative Medicine, Guangzhou Institutes of Biomedicine and Health, Chinese Academy of Sciences, Guangzhou 510530, China,; c Laboratory of RNA, Chromatin, and Human Disease, CAS Key Laboratory of Regenerative Biology and Guangdong Provincial Key Laboratory of Stem Cells and Regenerative Medicine, Guangzhou Institutes of Biomedicine and Health, Chinese Academy of Sciences, Guangzhou 510530, China,; d Cardiology Division, Department of Medicine, Queen Mary Hospital, The University of Hong Kong, Hong Kong SAR, China,; e Hong Kong-Guangdong Stem Cell and Regenerative Medicine Research Centre, The University of Hong Kong and Guangzhou Institutes of Biomedicine and Health, Hong Kong SAR, China,; f Department of Ophthalmology, The Third Affiliated Hospital of Sun Yat-sen University, Guangzhou 510630, China,; g State Key Laboratory of Organ Failure Research, Guangdong Provincial Key Laboratory of Viral Hepatitis Research, Guangdong Provincial Research Center for Liver Fibrosis, Department of Infectious Diseases and Hepatology Unit, Nanfang Hospital and; h Biomedical Research Center, Southern Medical University, Guangzhou 510515, China,; i Institute of Immunology and Immunotherapy, College of Medical and Dental Sciences,; j National Institute for Health Research (NIHR) Birmingham Liver Biomedical Research Unit and Centre for Liver Research, and; k Institute of Cancer and Genomic Sciences, College of Medical and Dental Sciences, University of Birmingham, Birmingham B15 2TT, United Kingdom,; l Cambridge Institute for Medical Research, Wellcome Trust/Medical Research Council (MRC) Building, Cambridge CB2 0XY, United Kingdom,; m Department of Clinical Biochemistry Unit, Queen Mary Hospital, Hong Kong SAR, China,; n Department of Medicine, University of Hong Kong-Shenzhen Hospital, Shenzhen 518053, Guangdong, China, and; o Laboratory of Metabolism and Cell Fate, CAS Key Laboratory of Regenerative Biology and Guangdong Provincial Key Laboratory of Stem Cells and Regenerative Medicine, Guangzhou Institutes of Biomedicine and Health, Chinese Academy of Sciences, Guangzhou 510530, Guangdong, China

**Keywords:** animal model, gene knock-out, liver, stem cells, transplantation

## Abstract

Hereditary tyrosinemia type 1 (HT1) is a severe human autosomal recessive disorder caused by the deficiency of fumarylacetoacetate hydroxylase (FAH), an enzyme catalyzing the last step in the tyrosine degradation pathway. Lack of FAH causes accumulation of toxic metabolites (fumarylacetoacetate and succinylacetone) in blood and tissues, ultimately resulting in severe liver and kidney damage with onset that ranges from infancy to adolescence. This tissue damage is lethal but can be controlled by administration of 2-(2-nitro-4-trifluoromethylbenzoyl)-1,3-cyclohexanedione (NTBC), which inhibits tyrosine catabolism upstream of the generation of fumarylacetoacetate and succinylacetone. Notably, in animals lacking FAH, transient withdrawal of NTBC can be used to induce liver damage and a concomitant regenerative response that stimulates the growth of healthy hepatocytes. Among other things, this model has raised tremendous interest for the *in vivo* expansion of human primary hepatocytes inside these animals and for exploring experimental gene therapy and cell-based therapies. Here, we report the generation of *FAH* knock-out rabbits via pronuclear stage embryo microinjection of transcription activator-like effector nucleases. *FAH*^−/−^ rabbits exhibit phenotypic features of HT1 including liver and kidney abnormalities but additionally develop frequent ocular manifestations likely caused by local accumulation of tyrosine upon NTBC administration. We also show that allogeneic transplantation of wild-type rabbit primary hepatocytes into *FAH*^−/−^ rabbits enables highly efficient liver repopulation and prevents liver insufficiency and death. Because of significant advantages over rodents and their ease of breeding, maintenance, and manipulation compared with larger animals including pigs, *FAH*^−/−^ rabbits are an attractive alternative for modeling the consequences of HT1.

## Introduction

Animal models bearing mutations/truncations in genes causing human disease are essential to understand the mechanisms underlying those diseases and to identify new diagnostic or therapeutic procedures. In some cases, the idiosyncrasy of human disease has also made these animals valuable for unexpected applications (1). One such example are *Fah*^−/−^ mice (2), which have been genetically engineered to lack fumarylacetoacetate hydrolase (FAH),^3^ an enzyme responsible for catalyzing the last step in the degradation of the amino acid tyrosine (3). In humans, lack of FAH causes hereditary tyrosinemia type 1 (HT1), a rare autosomal recessive condition characterized by retrograde accumulation of toxic metabolites including fumarylacetoacetate and succinylacetone in blood and tissues, which ultimately causes liver failure and renal tubular dysfunction as the most prominent manifestations (4, 5). Liver failure can develop acutely in the first few months after birth or be more progressive. Treatment of HT1 consists mainly of administration of 2-(2-nitro-4-trifluoromethylbenzoyl)-1,3-cyclohexanedione (NTBC), which inhibits the conversion of 4-hydroxyphenylpyruvate to homogentisic acid by 4-hydroxyphenylpyruvate dioxygenase, the second step in the tyrosine degradation pathway, thus preventing the accumulation of fumarylacetoacetate and succinylacetone (6). However, if patients are diagnosed too late or do not comply with NTBC treatment, there is end stage liver disease with cirrhosis and high risk of hepatocellular carcinoma due to chronic damage. Notably, in *Fah*^−/−^ mice, controlled withdrawal of NTBC can be used to cause liver damage in a desired manner, inducing the generation of a regenerative response that allows repopulation of the damaged liver by the remaining normal hepatocytes after NTBC is added back (2, 7). When *Fah*^−/−^ mice are simultaneously deficient in key genes regulating the immune response (*e.g. Rag2* and/or *Il2rg*), the same principle allows liver repopulation with transplanted heterologous primary hepatocytes (8), stem cell-derived hepatocytes (9), or transdifferentiated hepatocytes (10, 11) without immune rejection.

For a long time, genetic engineering of mammals has been mostly restricted to mice. This is because of the availability of mouse embryonic stem cells (ESCs) and the relative ease of manipulating these cells with traditional gene modification techniques. Genetically modified ESCs can then be injected into mouse blastocysts to produce chimeric animals with germ line transmission (12). Somatic cell modification and nuclear transfer have been traditionally an alternative for genetic engineering of species for which *bona fide* ESCs are not available, but the procedure is inefficient (13). More recently, the development of designer nuclease technologies (zinc finger nucleases, transcription activator-like effector nucleases (TALENs), and clustered regularly interspaced short palindromic repeats (CRISPR)/CRISPR-associated protein 9 (Cas9) (14) has expanded significantly the repertoire of species (*e.g.* rats, rabbits, dogs, pigs, sheep, and cattle) (15–20) amenable to routine genetic engineering and made the procedure a less time-consuming effort; *bona fide* rat ESCs were also isolated recently (21, 22). Accordingly, several groups have reported the generation of FAH-deficient pigs (23, 24) and rats (25, 26), which offer some advantages over *Fah*^−/−^ mice.

Rabbits are widely used for animal experimentation. Their physiology is closer to humans than rodents, and they have relatively low cost maintenance and are easy to breed (27, 28). Herein, we report the generation of *FAH*^−/−^ rabbits by injecting TALENs into the cytoplasm of rabbit pronuclear stage embryos. These *FAH*^−/−^ rabbits have liver and kidney phenotypic features analogous to FAH-deficient rodents and pigs but also develop frequent ocular manifestations including corneal keratitis. We further show that allogeneic transplantation of wild-type rabbit primary hepatocytes achieves efficient liver repopulation and improves the liver function and survival rate of *FAH*^−/−^ rabbits. These results demonstrate that genetically engineered *FAH*^−/−^ rabbits are an attractive choice for modeling the consequences of HT1.

## Results

### 

#### 

##### Construction of TALENs and Generation of FAH Knock-out Rabbits

**FIGURE 1.**
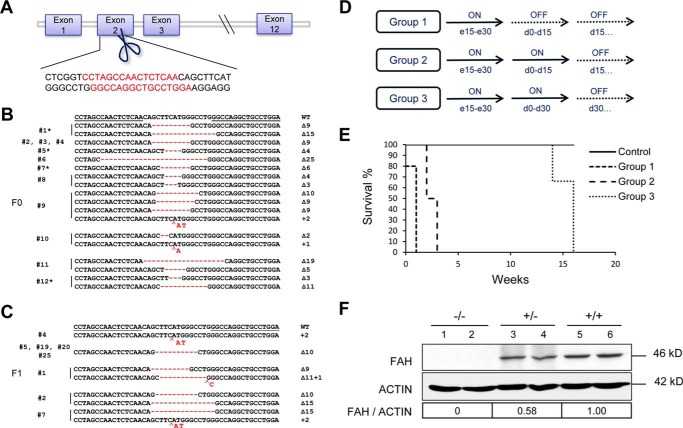
**Preparation of TALENs and generation of *FAH* knock-out rabbits.** *A*, design of TALENs targeting exon 2 of rabbit *FAH* gene. Bases in *red* indicate the TALEN recognition sequences. *B*, Sanger sequencing of the targeted region in the *FAH* locus in F0 rabbits; * represents rabbits in which the wild-type *FAH* sequence was detected. *C*, Sanger sequencing of the targeted region in the *FAH* locus in *FAH*^−/−^ F1 rabbits. *D*, schematic of the three groups of *FAH*^−/−^ rabbits used to study the dependence on NTBC for long term survival (*ON*, NTBC administration; *OFF*, NTBC withdrawal); *e* means embryonic day and *d* means day after birth. *E*, survival curve for the same three groups of *FAH*^−/−^ rabbits. *F*, Western blotting analysis confirms that *FAH*^−/−^ rabbits (−/−) are negative for FAH protein expression in liver tissue lysates, whereas *FAH*^+/−^ (+/−) rabbits express a reduced amount of FAH. Band intensities (FAH/Actin) were quantified using ImageJ software and are shown relative to wild-type (+/+) rabbits.

We designed a pair of TALENs targeting exon 2 of the rabbit *FAH* gene (Fig. 1*A*) and assembled them according to the Golden Gate method with the following codes: NI for adenine, NG for thymine, HD for cytosine, and NN for guanine. We injected different concentrations (10, 20, 30, 50, and 100 ng/μl) of *in vitro* transcribed TALEN-coding mRNAs into the cytoplasm of rabbit pronuclear stage embryos (20, 27) and then transferred the embryos into surrogate mothers. NTBC was administered to pregnant rabbits from day 15 of pregnancy to prevent intrauterine death (2). With the highest concentration of TALEN mRNAs, all foster mothers miscarried, and no rabbits were born (Table 1). The pregnancy was not affected with the other TALEN mRNA concentrations; a total of 31 rabbits were born, and the ear tissue of each animal was collected for genotyping. With the lowest concentration of TALEN mRNAs, all six newborn rabbits were negative for gene targeting. However, we detected *FAH* mutations using 20 and 30 ng/μl, and the targeting efficiency was 100% (four of four newborns) using 50 ng/μl (Table 1). These *FAH* mutant rabbits were mostly mosaic with different insertions and/or deletions (indels) within the *FAH* locus as shown in (Fig. 1*B*). Through breeding of *FAH* mutant founder (F0) animals, we obtained a total of nine *FAH*^−/−^ first filial generation (F1) rabbits (Fig. 1*C*) and eight *FAH*^+/−^ rabbits, which appeared healthy at birth. These animals were bred further to produce additional filial generations.

**TABLE 1:** **Generation of *FAH* knock-out rabbits using TALENs**

No. surrogates	TALEN mRNA concentration	No. embryos transferred	No. newborns (%)	No. mutant rabbits (%)
	*ng*/μ*l*			
3	10	34	6 (12)	0
3	20	39	12 (31)	3 (25)
2	30	32	9 (28)	5 (56)
3	50	37	4 (11)	4 (100)
4	100	40	0	0

To ascertain the importance of NTBC administration in preventing death of *FAH*^−/−^ rabbits, we prepared 13 *FAH*^−/−^ rabbits and divided them into three groups, each with different NTBC administration modes (Fig. 1*D*). The rabbits were closely monitored to collect blood before death and to store the tissues in good condition. In the first group, no NTBC was administered at any time after birth, and these animals died shortly (within 1 week after birth) (Fig. 1*E*). In the second and third groups, NTBC was continuously administered until the rabbits were 2 weeks or 1 month old, respectively. Rabbits in the second group survived no longer than 3 weeks after birth, whereas rabbits in the third group were able to live as long as 4 months after birth (Fig. 1*E*). Western blotting analysis of liver tissue lysates from two dead *FAH*^−/−^ rabbits showed that FAH was undetectable when compared with wild-type rabbits (Fig. 1*F*). Likewise, heterozygous knock-out (*FAH*^+/−^) rabbits expressed half of the amount of protein of the wild type. Thus, we have generated *FAH*^−/−^ rabbits that can only be maintained alive with NTBC, and we next proceeded to study whether these animals develop characteristic phenotypic features of HT1.

##### FAH^−/−^ Rabbits Have Liver and Kidney Phenotypic Characteristics of HT1

**FIGURE 2.**
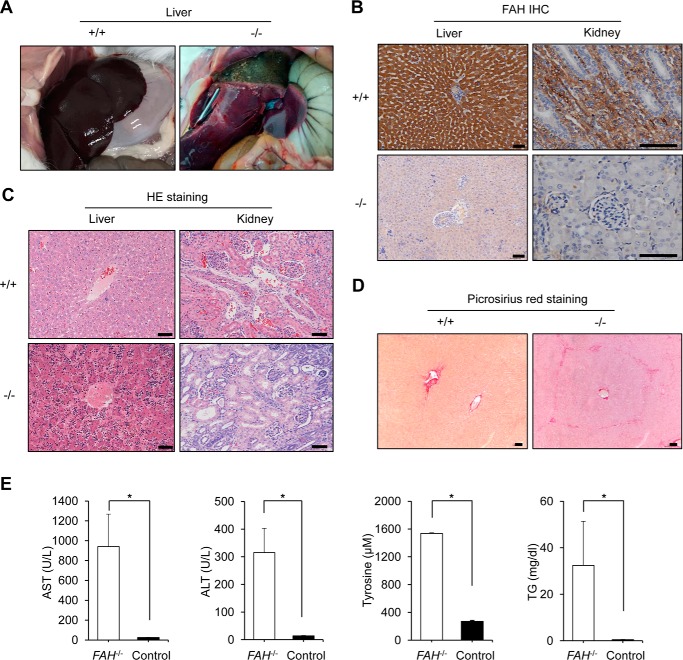
***FAH*^−/−^ rabbits develop progressive liver failure in the absence of NTBC.** *A*, severe necrosis in the liver of a *FAH*^−/−^ rabbit (*right*) belonging to group 3 of NTBC administration (as in Fig. 1*D*) in contrast to a healthy wild-type rabbit. *B*, immunohistochemistry (*IHC*) of liver and kidney sections of a *FAH*^−/−^ rabbit belonging to group 2 of NTBC administration shows no expression of FAH in contrast to a wild-type rabbit. *Scale bars*, 50 μm. *C*, hematoxylin and eosin (*HE*) staining shows abnormal tissue architecture in liver and kidney sections of the same *FAH*^−/−^ rabbit in *B* in contrast to a wild-type rabbit. In the *FAH*^−/−^ rabbit, diffused hepatocellular injury with dysplastic hepatocytes and tubular epithelial injury of kidney were observed. *Scale bars*, 50 μm. *D*, picrosirius red staining shows the existence of mild interstitial fibrosis in the liver of the same *FAH*^−/−^ rabbit in *A* but not in the wild-type rabbit. *Scale bars*, 50 μm. *E*, serum biochemical parameters indicate liver damage in *FAH*^−/−^ rabbits compared with a wild-type rabbit. *TG* stands for triglycerides. *Error bars* represent S.E. (*n* = 3 replicate measurements). * corresponds to *p* < 0.01 according to Student's *t* test.

We dissected the bodies of dead *FAH*^−/−^ rabbits belonging to the above mentioned groups and observed prominent liver swelling, hemorrhage, and yellow/green discoloration suggestive of both cholestasis and liver necrosis in contrast to the normal aspect of their wild-type counterparts (Fig. 2*A*). We also collected and sectioned the liver and kidney tissues for immunohistological analysis with FAH antibodies. As expected, FAH protein was completely absent in *FAH*^−/−^ livers and kidneys, two tissues that normally express it at a high level, but displayed strong staining in wild-type rabbits (Fig. 2*B*). In addition, hematoxylin and eosin staining showed diffuse hepatocellular injury with dysplastic hepatocytes and tubule interstitial nephritis in *FAH*^−/−^ rabbits only (Fig. 2*C*), both of which are hallmarks of HT1. Moreover, picrosirius red staining (29) revealed mild fibrosis in the liver of *FAH*^−/−^ rabbits but not in wild type (Fig. 2*D*), which is in agreement with *Fah*^−/−^ mice but contrasts with the development of cirrhosis in FAH-deficient rats and pigs (25, 30). In addition, we extracted serum from blood samples and analyzed the levels of alanine aminotransferase (ALT), aspartate aminotransferase (AST), and triglycerides, all of which are elevated as a consequence of liver damage (23). A significant increase of all three parameters was observed in *FAH*^−/−^ rabbits maintained without NTBC compared with wild-type rabbits (Fig. 2*E*). We also detected that tyrosine levels had increased in *FAH*^−/−^ rabbits maintained without NTBC, illustrating that, as expected, deletion of *FAH* blocks the tyrosine metabolic pathway (2, 5). Altogether, these results confirm that *FAH*^−/−^ rabbits develop features similar to HT1 patients and rodent/pig models (5, 7, 23–26).

##### Ocular Manifestations in FAH Knock-out Rabbits

**FIGURE 3.**
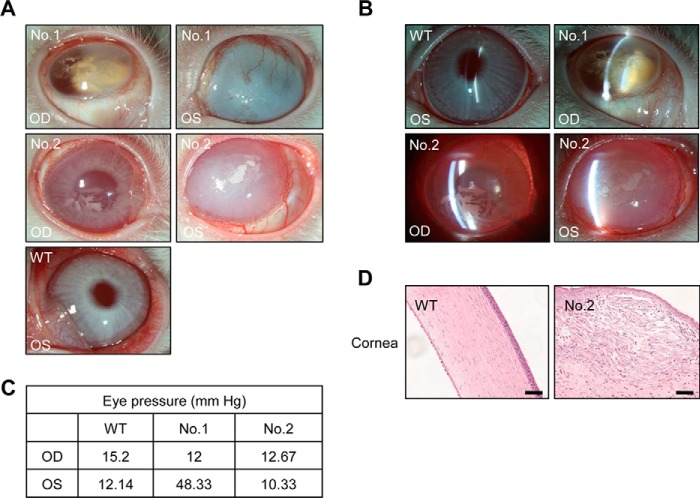
**Ocular manifestations in *FAH* knock-out rabbits.** *A*, photographs of right (*OD*) and left (*OS*) eyes of two *FAH*^+/−^ rabbits show corneal abnormalities; a wild-type (*WT*) rabbit was used as control. *B*, slit lamp photographs of the same three rabbits show abnormalities of chamber depth and lens opacification in *FAH*^+/−^ rabbit number 1 compared with the wild type. *C*, measurement of intraocular pressure using a tonometer (Icare, Finland) in the same three rabbits. Only the left eye of *FAH*^+/−^ rabbit number 1 shows pathogenic levels. *D*, hematoxylin and eosin staining shows corneal structural abnormalities (edema, thickening, cellular defects, and disorganization of the corneal epithelium and stroma) in the left eye of *FAH*^+/−^ rabbit number 2 compared with the wild type. *Scale bars*, 50 μm.

Ocular involvement is not frequent in HT1 patients, but in those rare cases corneal keratitis is the main manifestation (31, 32). This has been attributed to inflammation produced by local tyrosine deposition in the form of crystals caused by low compliance with a low protein diet and the secondary effect of using NTBC on tyrosine accumulation. Interestingly, in the course of our study, we noticed that *FAH* knock-out rabbits develop frequent ocular manifestations too. This problem was observed in all third filial generation *FAH*^−/−^ rabbits and a small proportion of *FAH*^+/−^ rabbits. Of note, although the latter were not treated with NTBC at any time after birth, it was administered to their mothers during pregnancy. We performed ophthalmological analysis of two *FAH*^+/−^ rabbits compared with a wild-type rabbit. Through direct eye inspection, we discovered keratoleukoma accompanied by edema and opacity in both eyes of the two *FAH*^+/−^ rabbits (Fig. 3*A*). Using slit lamp imaging, we also noticed deeper chamber depth and lens opacification accompanied with dilated pupil and posterior synechia of the iris in the right eye of the first *FAH*^+/−^ rabbit (Fig. 3*B*), implying cataract and iritis. Moreover, in the same rabbit, there was a pathogenic high intraocular pressure of 48.33 mm Hg in the left eye (Fig. 3*C*), indicating secondary glaucoma, whereas intraocular pressure in the right eye was normal. Conversely, chamber depth and intraocular pressure were normal in the second *FAH*^+/−^ rabbit (Fig. 3, *B* and *C*), and no significant cataract was found. In addition, hematoxylin and eosin staining showed edema and thickening in the corneal epithelium and stroma of both *FAH*^+/−^ rabbits, and the corneal surface became irregular in particular in the left eye of the second *FAH*^+/−^ rabbit (Fig. 3*D*). Moreover, swelling, condensed nuclei, and fragmented bodies could be seen in corneal epithelial and stroma cells in both *FAH*^+/−^ rabbits, indicating necrocytosis (Fig. 3*D*). Therefore, there is frequent ocular involvement, mostly manifested as corneal keratitis, in *FAH* knock-out rabbits.

##### Allogeneic Primary Hepatocyte Transplantation Rescues Liver Damage of FAH^−/−^ Rabbits

**FIGURE 4.**
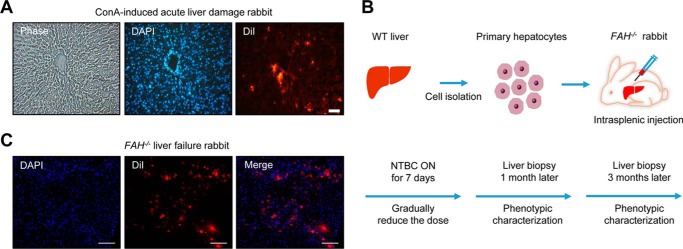
**Hepatocyte transplantation of *FAH*^−/−^ rabbits.** *A*, transplantation of wild-type rabbit primary hepatocytes into the liver of a wild-type rabbit treated with concanavalin A (*ConA*; 5.0 mg/kg, intrasplenic injection) to induce acute liver damage. Engraftment of DiI-labeled hepatocytes can be observed under the fluorescence microscope at 3 weeks post-transplantation. DAPI was used to stain nuclei. *Scale bars*, 50 μm. *B*, schematic showing the allogeneic transplantation procedure in *FAH*^−/−^ rabbits. NTBC was administered for another 2 days after the first liver biopsy. *C*, engraftment of DiI-labeled cells observed under the fluorescence microscope in a *FAH*^−/−^ rabbit without NTBC at 1 month post-transplantation. *Scale bars*, 50 μm.

**FIGURE 5.**
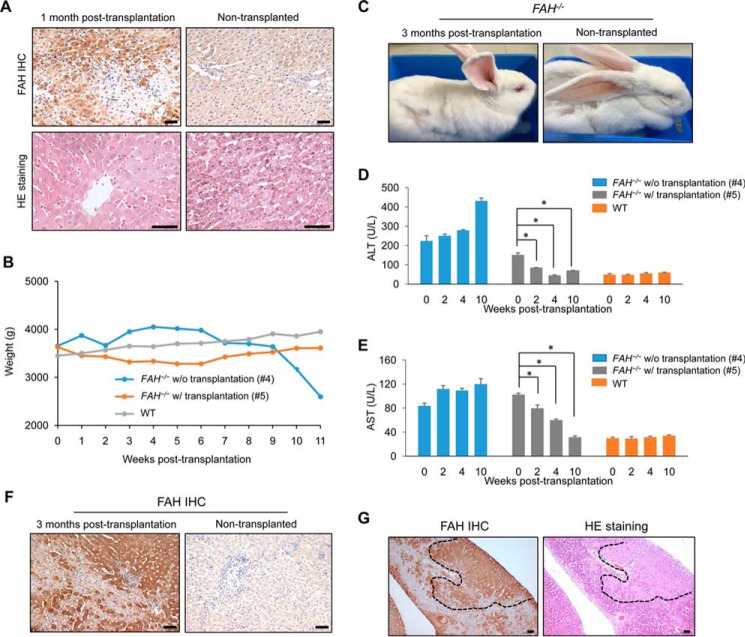
**Wild-type primary hepatocyte transplantation rescues liver architecture and prevents death of *FAH*^−/−^ rabbits.** *A*, *top panels*, FAH immunohistochemistry shows wild-type hepatocyte repopulation of the liver of a *FAH*^−/−^ rabbit 1 month post-transplantation compared with the control (non-transplanted *FAH*^−/−^ rabbit). *Lower panels*, hematoxylin and eosin staining shows restoration of liver architecture in the same transplanted *FAH*^−/−^ rabbit compared with the non-transplanted *FAH*^−/−^ rabbit. *Scale bars*, 50 μm. *B*, body weight changes of the two *FAH*^−/−^ rabbits in *A* (with or without transplantation) and a control wild-type rabbit. * corresponds to *p* < 0.01. *C*, photographs of a transplanted *FAH*^−/−^ rabbit at 3 months post-transplantation and a non-transplanted *FAH*^−/−^ rabbit. The non-transplanted *FAH*^−/−^ rabbit appears weak. *D* and *E*, serum levels of ALT and AST in a wild-type rabbit and two *FAH*^−/−^ rabbits (with (*w/*) or without (*w/o*) transplantation). *Error bars* represent S.E. (*n* = 3 replicate measurements). * corresponds to *p* < 0.01. *F*, FAH immunohistochemistry shows extensive wild-type hepatocyte repopulation of the liver of a *FAH*^−/−^ rabbit 3 months post-transplantation compared with the non-transplanted *FAH*^−/−^ rabbit. *Scale bars*, 50 μm. *G*, *right panel*, hematoxylin and eosin (*HE*) staining shows restoration of liver architecture (*circled* by *dashed lines*) in the same transplanted *FAH*^−/−^ rabbit at 3 months post-transplantation. *Left panel*, adjacent liver section of the transplanted *FAH*^−/−^ rabbit showing co-localization of areas with normal structure and positive FAH staining. *Scale bars*, 50 μm.

We then studied whether transplantation of *FAH*-competent hepatocytes could rescue the liver phenotype and prevent death of *FAH*^−/−^ rabbits untreated with NTBC. First, to assess feasibility, we induced acute liver damage in wild-type rabbits with concanavalin A (33), which causes a rapid regenerative response that facilitates engraftment of transplanted cells, and 24 h later 10^7^ rabbit primary hepatocytes extracted from a healthy wild-type rabbit were injected intrasplenically. To assist with their identification, transplanted hepatocytes were labeled with DiI, as this fluorescent compound can last as long as 1 month *in vivo* (34, 35). The recipient animals were immunosuppressed with cyclosporin A starting 24 h before transplantation. We observed a large number of DiI-positive cells in liver sections 3 weeks after transplantation, proving the efficacy of the approach (Fig. 4*A*). Next, we transplanted wild-type rabbit primary hepatocytes into two *FAH*^−/−^ rabbits that had been treated with NTBC after birth. A wild-type rabbit and a non-transplanted *FAH*^−/−^ rabbit treated with NTBC were used as controls. NTBC was gradually decreased in the three *FAH*^−/−^ rabbits and completely withdrawn 1 week after the transplantation (Fig. 4*B*). One month after NTBC withdrawal, a small liver biopsy was obtained from one of the transplanted *FAH*^−/−^ rabbits and the non-transplanted *FAH*^−/−^ rabbit for histological analysis. Approximately 30% of liver cells in the examined liver sections of the transplanted *FAH*^−/−^ rabbit were DiI-positive at this time point (Fig. 4*C*). Likewise, FAH immunohistochemistry confirmed significant engraftment of FAH-positive hepatocytes in the same *FAH*^−/−^ transplanted rabbit, and these hepatocytes displayed normal morphology in contrast with damaged cells in non-engrafted areas (Fig. 5*A*). We also measured weight in these *FAH*^−/−^ rabbits and the wild-type control over the post-transplantation period. We noticed that 11 weeks after NTBC withdrawal the *FAH*^−/−^ rabbit that did not receive transplantation showed a 40% decrease in body weight and died shortly after, whereas the wild-type and *FAH*^−/−^ rabbits receiving allogeneic hepatocyte transplantation had similar weight and appeared healthy (Fig. 5, *B* and *C*). In addition, we performed serum biochemical analysis over the post-transplantation period to see whether liver function can be recovered in *FAH*^−/−^ rabbits compared with the control. Indeed, ALT and AST values of wild type and a transplanted *FAH*^−/−^ rabbit were alike at week 10 post-transplantation, whereas the non-transplanted *FAH*^−/−^ rabbit showed significantly higher values (Fig. 5, *D* and *E*). Interestingly, we also observed more engrafted FAH-positive cells at 3 months in the transplanted *FAH*^−/−^ rabbits (Fig. 5, *F* and *G*), indicating long term stability of the engrafted cells and suggesting *in vivo* proliferation. These data prove the utility of *FAH*^−/−^ rabbits as tools for testing experimental methodologies involving liver cell transplantation.

## Discussion

Patients with HT1 are treated with NTBC and dietary restrictions, but these are not curative, and, besides, a number of individuals fail to respond and require liver transplantation (7). Because a shortage of donors limits organ transplantation, other therapeutic strategies (*e.g.* stem cell-based or gene therapy approaches) are urgently needed to treat this patient population. Appropriate preclinical animal models are required for testing these experimental therapies, and although *Fah*^−/−^ mice present many phenotypic features of HT1, their physiology (*e.g.* inflammatory responses (36)) differs significantly from humans. Likewise, their small size and short life span pose a limitation for analytical studies and long term assessments. Aiming to solve these issues, *Fah*^−/−^ rats (25, 26) and in particular pigs (23, 24) have been generated recently. However, although pigs have many advantages over rodents for HT1 disease modeling, their handling and breeding is laborious and costly.

Rabbits are excellent animals for state-of-the-art animal experimentation. They are closer phylogenetically to primates than rodents and have a longer life span (8–10 years), and their medium size, short pregnancy period (1 month *versus* 4 months in pigs), and relatively straightforward husbandry requirements facilitate production of large cohorts at relatively low cost (27, 28). Like pigs, rabbits also have a more diverse genetic background than rodents, a situation that is closer to that in humans. Notably, the first transgenic rabbits were generated over 3 decades ago (37, 38), but the lack of *bona fide* rabbit ESCs for more complex genetic engineering and the inefficiency of rabbit somatic cell nuclear transfer (39) hampered the development of the field until the arrival of highly efficient designer nuclease technologies (20, 40–42).

Our work presented here is the first description of genetically engineered *FAH*^−/−^ rabbits. We used TALEN mRNA microinjection into pronuclear stage rabbit embryos (20) rather than the CRISPR/Cas9 system (40, 43) because we envisaged that for embryo injections the toxicity and off-target effects of the former are easier to control (44), although recent improvements of the CRISPR/Cas9 technique could solve this issue (45). *FAH*^−/−^ rabbits display liver and kidney manifestations of the human genetic disorder but also have frequent ocular alterations (mostly corneal keratitis). Ocular involvement is rare in patients with HT1 but frequent in patients with tyrosinemia type II (46), which is produced by mutations in tyrosine transaminase, the enzyme undertaking the first step of tyrosine catabolism. As with tyrosinemia type II, corneal keratitis in HT1 patients and possibly *FAH* knock-out rabbits as well is caused by enhanced local accumulation of tyrosine, which is boosted by NTBC (31, 32). The frequency with which ocular manifestations happen in *FAH*^−/−^ rabbits potentially makes them a useful model for studying how to prevent and treat this potential complication in HT1 patients. *FAH*^−/−^ rabbits did not develop cirrhosis in our study, although it is likely that varying the NTBC administration/withdrawal routine and allowing more chronic damage would induce it. Besides being an excellent choice for modeling HT1 and studying chronic liver damage, *FAH*^−/−^ rabbits could also be used for testing gene therapy approaches, which have proved successful in FAH-deficient mice and pigs but require additional studies to evaluate long term safety and efficacy (24). In addition, *FAH*^−/−^ rabbits could be exceptional bioreactors for growing primary human hepatocytes for *in vivo* human disease modeling (*e.g.* hepatitis B or C), potential xenotransplantation, or for *in vitro* studies, but this would require producing *FAH*^−/−^*RAG2*^−/−^*IL2RG*^−/−^ rabbits to avoid immune rejection. Such triple knock-out animals would be valuable too as preclinical models for experimental stem cell-based therapies including the transplantation of hepatocyte-like cells derived from induced pluripotent stem cells (11) or produced through transdifferentiation (10, 11). Given the time and cost of pursuing this approach in pigs, rabbits offer an attractive option.

In summary, we have demonstrated that *FAH*^−/−^ rabbits are a promising alternative for modeling HT1 and for developing therapeutic strategies aiming to cure this disease. From a wider perspective, our work also shows that genetically engineered rabbits offer a powerful approach to recapitulating human disease.

## Experimental Procedures

### 

#### 

##### Animals and Ethics Statement

New Zealand White rabbits were obtained from the Laboratory Animal Centre of Southern Medical University (Guangzhou, China). All rabbit experiments were conducted under the approval of the Animal Care and Use committee of the Guangzhou Institutes of Biomedicine and Health (ID 2012040) and the Department of Science and Technology of Guangdong Province (ID SYXK 2005-0063). Animals were observed at least once daily for clinical signs and symptoms consistent with acute liver failure. To obtain *FAH*^−/−^ and *FAH*^+/−^ F1 rabbits, we bred F0 animals with each other, and then we bred F0 with F1, F1 with F1, and so on for producing other filial generations. For details of which rabbits were used in each experiment and their respective genotypes see supplemental Table 1. Pregnant rabbits giving birth to *FAH*^−/−^ rabbits received 7.5 mg/liter NTBC/200 ml of drinking water per day beginning on day 15 of pregnancy. The same dose was used for maintenance of *FAH*^−/−^ rabbits. Ophthalmological evaluation was performed in Zhongshan Ophthalmic Center, Sun Yat-sen University.

##### TALEN Preparation, Embryo Microinjection, and Embryo Transfer

TALENs were designed and assembled according to the golden gate assembly method (27). *In vitro* transcribed TALEN mRNAs were prepared using an mMESSAGE mMACHINE® T7 kit (Ambion, Austin, TX) and purified using an RNeasy Mini Elute Cleanup kit (Qiagen, Valencia, CA). The TALEN microinjection procedure was essentially as described previously (27). Briefly, 6–8-month-old female rabbits were induced to superovulate with 50 IU of FSH every 12 h for 3 days, mated with male rabbits about 72 h later, and injected with 100 IU of human chorionic gonadotropin. Female rabbits were euthanized 18 h after injection, and the oviducts were flushed with prewarmed embryo manipulation medium for collecting embryos at the pronuclear stage. Mixed *in vitro* transcribed TALEN mRNAs were microinjected into the cytoplasm, and then embryos were transferred to Earle's balanced salt solution medium for *in vitro* culture in a 5% CO_2_ incubator at 38.5 °C with 100% humidity. Approximately 15–30 optimal quality (as judged by microscope inspection) injected embryos were transferred into unilateral pavilions of the oviducts for each recipient mother.

##### Genotyping

DNA of newborn rabbits was extracted from a small piece of ear tissue using a Hipure Tissue DNA Mini kit (Magen, Beijing, China) following the manufacturer's protocol. PCR products spanning the TALEN target sites were amplified with KOD-Plus-Neo DNA polymerase (Toyobo, Tokyo, Japan) with the primers 5′-GCACTTGAGCCATCGTCCGT (FAH-F) and 5′-ACCAGCAGCAGGCAATCCCA (FAH-R) and then sequenced.

##### Western Blotting Assay

For Western blotting, liver tissue samples were homogenized in radioimmune precipitation assay buffer and centrifuged at 14,500 × *g* at 4 °C for 30 min; supernatants were then collected, and protein concentration was quantified. Lysates were subjected to 10% polyacrylamide-SDS gel electrophoresis followed by immunoblotting onto a polyvinylidene fluoride membrane (Millipore, Temecula, CA). Membranes were blocked with 5% evaporated milk in TBS-Tween 20 for 2 h and incubated overnight at room temperature with primary antibodies against FAH (ab140167, Abcam, Cambridge, UK). Membranes were then incubated with a secondary HRP-conjugated anti-rabbit antibody (sc-2004, Santa Cruz Biotechnology, Santa Cruz, CA) for 60 min at room temperature and imaged using a SuperSignal^TM^ West Pico Chemiluminescence Substrate kit (Thermo, Rockford, IL). ACTIN antibody (sc-47778, Santa Cruz Biotechnology) was probed as a loading control.

##### Histology

Liver and kidney tissues were fixed with 4% neutral buffered paraformaldehyde for 48 h and processed for paraffin embedding and sectioning. For immunohistochemistry staining, antigen retrieval was performed in a 1 m citrate buffer (pH 6.0) bath for 20 min. Tissues were immunostained with anti-FAH primary antibodies (ab140167) and visualized using VECTASTAIN Elite ABC HRP kit (Vector Laboratories, Burlingame, CA). Hematoxylin and eosin staining and picrosirius red staining were performed using standard protocols.

##### Serum Analysis

Blood was obtained via puncture of the ear vein using heparinized tubes. Serum was separated by centrifugation at 900 × *g* for 20 min and stored at −80 °C prior to biochemistry and amino acid analysis. Concentrations of ALT, AST, and triglycerides were measured with a CL-8000 Hitachi 7180 automatic biochemical analyzer (Shimadzu, Kyoto, Japan). Tyrosine was quantified by liquid chromatography-mass spectrometry using the ABI 3200 Q TRAP LC-MS/MS system (Applied Biosystems, Foster City, CA).

##### Allogeneic Hepatocyte Transplantation

Fresh hepatocytes were isolated from wild-type rabbit livers by *in situ* collagenase perfusion as reported previously (47). Briefly, the liver was perfused with calcium- and magnesium-free Hanks' balanced salt solution (Thermo) supplemented with 0.5 mm EGTA for 10 min and 10 mm HEPES for 3 min. The solution was changed to Earle's balanced salt solution supplemented with 0.1 mg/ml collagenase IV (Sigma) for 10 min. The liver was then gently minced in the second solution and filtered through 150-, 75-, 50-, and 37.5-μm nylon mesh, sequentially. After centrifugation at 50 × *g* for 5 min, the pellet was washed with DMEM Gibco) supplemented with 4.5 g/ml glucose three times at 50 × *g* for 2 min. The number and viability of cells were assessed by trypan blue. 10^7^ viable cells were then incubated in a 20 μm DiI solution (Sigma) for 5 min at 37 °C and for 15 min at 4 °C for labeling and washed in DMEM supplemented with 4.5 g/ml glucose. Eventually, 10^7^ hepatocytes in 2.5 ml of Clonetics® hepatocyte culture medium (Lonza, Walkersville, MD) were injected into the spleen of recipient rabbits via a small flank incision. Concanavalin A and cyclosporin A were purchased from Sigma.

## Author Contributions

M.A.E. and L. Lai had the idea for the study. L. Li, L. Lai, and M.A.E. designed the experiments. L. Li and M.A.E. analyzed the data. L. Li conducted most of the experiments. Q. Zhang, H. Y., Q. Zou, C. L., F. J., P. Z., Z. L., J. Y., Q. C., Y. W., P. N. N., J. F., P. H. M., W. L., S. C., D. W., T.-K. S., S. T., H.-F. T., B. Q., and X. B. contributed to the experiments or provided critical advice. M. A. E., X. B., and L. Lai provided funding. M. A. E. wrote the manuscript with help from L. Li. M. A. E. and L. Lai approved the final version of the manuscript for submission.

## Supplementary Material

Supplemental Data

## Footnotes

3 The abbreviations used are:

FAH: fumarylacetoacetate hydroxylase
ALT: alanine aminotransferase
AST: aspartate aminotransferase
CRISPR: clustered regularly interspaced short palindromic repeats
Cas9: CRISPR-associated protein 9
DiI: 1,1-dioctadecyl-3,3,3,3-tetramethylindocarbocyanine perchlorate
ESCs: embryonic stem cells
F0: founder generation
F1: first filial generation
HT1: hereditary tyrosinemia type 1
NTBC: 2-(2-nitro-4-trifluoromethylbenzoyl)-1,3-cyclohexanedione
TALEN: transcription activator-like effector nuclease.
