# Supplementary material for: Fumarylacetoacetate Hydrolase Knock-out Rabbit Model for Hereditary Tyrosinemia Type 1
Source: J Biol Chem. 2017 Jan 4;292(11):4755–63. doi: 10.1074/jbc.M116.764787 (PMC5377789; doi:10.1074/jbc.M116.764787)
Supplement: Supplemental Data [file supp_292_11_4755__index.html]

Fumarylacetoacetate hydrolase knockout rabbit model for hereditary tyrosinemia type 1 — Fumarylacetoacetate Hydrolase Knock-out Rabbit Model for Hereditary Tyrosinemia Type 1 — Fumarylacetoacetate Hydrolase Knock-out Rabbits — Supplemental Data 

# Fumarylacetoacetate Hydrolase Knock-out Rabbit Model for Hereditary Tyrosinemia Type 1

## Supplemental Data

- Supplemental Table I (.pdf, 23 KB) - Genotyping results for rabbits in all Figures
