# Supplementary material for: Fumarylacetoacetate Hydrolase Knock-out Rabbit Model for Hereditary Tyrosinemia Type 1
Source: J Biol Chem. 2017 Jan 4;292(11):4755–63. doi: 10.1074/jbc.M116.764787 (PMC5377789; doi:10.1074/jbc.M116.764787)
Supplement: Supplemental Data [file 10.1074_M116.764787_jbc.M116.764787-1.pdf]

Supplemental Table I

Figure	Genotype	No. of rabbits	Generation
1D and E, group 1	(+2, +2), (+2, -15), (-10, -10)	3	F1
1D and E, group 1	(+2, -9), (-9, -11+1), (-10, -15)	3	F2
1D and E, group 2	(+2, +2), (-10, -10), (+2, -15), (-10, -9)	4	F1
1D and E, group 3	(-10, -10)	3	F2
1F, (-/-)	(+2, +2)	1	F1
1F, (+/-)	(+2, WT), (-10,WT)	2	F1
2A and D, (-/-)	(-10, -10)	3	F2
2B and C, (-/-)	(+2, +2), (-10, -10), (+2, -15)	3	F1
2E, (-/-)	(-10, -10), (+2, -15)	2	F1
3, No. 1	(-10, WT)	1	F2
3, No. 2	(+2, WT)	1	F3
4C	(-10, -10)	1	F1
5, transplanted (-/-)	(-10, -10), (+2, -15)	2	F1
5, non-transplanted (-/-)	(+2, +2)	1	F1
